# Case of Puerperal Convulsions

**Published:** 1827-08

**Authors:** G. Jones


*Case of Puerperal Convulsions.* By G. Jones, M.R.C.S. L.

Mrs. S—, æt. twenty-three, of a full plethoric habit, ruddy complexion, and frequently the subject of headache, was taken in labour with her first child on the morning of the 24th January. At four P.M., the os uteri not being dilated to more than the size of a half-crown piece, the membranes gave way, and the liquor amnii was evacuated. Severe labour-pains continued, with but little interruption, until seven o'clock, at which time the head was full in the pelvis, and resting upon the perineum, when she was seized with a convulsive paroxysm. The belly became rigidly extended; a frothy saliva, tinged with blood, (occasioned by biting her tongue,) issued from her mouth, accompanied with stertorous breathing. I immediately opened a vein in the arm, and allowed the blood to flow until a sensible impression was made upon the system. I then prepared to deliver my patient with the forceps, when the uterine action, which had been suspended for the space of half an hour, returned, and delivery was speedily effected by the natural efforts. The placenta came away in the usual manner, and without any particular discharge. After the lapse of a short time, she was attacked with a second fit, and I administered the following draught—

℞. Tinct. Opii gtt. xxx.; Sp. Æther. Sulph. ʒj.; Mist. Camph. ℥jss. M. fiat haustus.

She expressed herself relieved. The pulse was full, strong, and frequent, and her face much flushed. After directing the head and face to be kept constantly wetted with a cold cloth, I left her. Her husband called me up at two A.M. saying his wife had had seven fits since I saw her, and of greater violence. I tied up the arm, and bled her a second time, and dropped a full dose of croton-oil into her mouth. The convulsions increasing in violence and frequency after a short interval, I gave her the following powder—

℞. Hydrarg. Subm. ℈ss.; Acet. Morphiæ gr. ss. M. fiat pulvis.

This also was administered by dropping into the mouth, for between the paroxysms she lay in a comatose state, and quite unconscious of those about her, with a great disinclination to be roused. I now retired for the night, after having desired the cold applications to be continued to the head, and some Camphor mixture given as often as it could be taken; wishing to see the effect of the calomel and croton-oil upon the bowels, before any other active measures were resorted to.

25th.—This morning the nurse informs me that my patient has passed a miserable night, having had seventeen fits; she is totally insensible to surrounding objects; the pupil is singularly contracted; the pulse 120; the countenance much flushed. The bowels not having acted, I directed an assafœtida glyster, which speedily relieved them.

Applic[r] Empl. Canth. inter scapulas. Applic[r] Lotio spirit. capiti raso.

It became quite impossible to administer any medicine of a bulky form.

Two P.M.—The symptoms continue without intermission or remission. From the very contracted state of the pupil, and from witnessing the good effects of belladonna in a similar case, I prescribed a grain of the extract to be taken every four hours.

Eight P.M.—Has had no fit for an hour and a half. Bowels have acted freely. Coma still continues; pulse 120.

Cont[r] Ext. Belladonnæ. Applic[r] Sinapism pediluv.

26th.—Has passed a more tranquil night, and free from convulsions. The blister has risen, but the sinapisms have not acted. Pulse 120, much softer; she is roused with much difficulty; bowels much relaxed; lochia as usual. She has taken in the whole six grains of the Extract of Belladonna, and with manifest advantage.

Omitt[r] Ext. Belladonnæ. Cont[r] Lotio. Repet[r] *S*inapism.—℞. Tinct. Hyosc. ʒss.; Mist. Camph. ℥j. M. fiat haustus quartâ quâque horâ sum.

Vesperi.—Much wildness of the eyes; coma quite gone; countenance much flushed. She takes more notice of those about her, but without speaking. Pulse 130, weak. The sinapisms have acted.

Cont[r] medicamenta.

27th.—This morning, for the first time, she answers my questions rationally. Complains much of her head; pulse weak, and rather fluttering; bowels quite relaxed.

Applic[r] Hirudines xij. capiti raso.—℞. Conf. Aromat. ʒj.; Mist. Camp. ℥vj. M. coch. larg. iij. quartâ quâque horâ sumend.—Cont[r] Lotio.

Vesperi.—Has been delirious during part of the day, and answers very incoherently. The same wildness of the eyes.

28th.—No sleep during the whole of the night. Much prostration of strength; pulse frequent and tremulous. She complains much of her head being light, and of soreness of her tongue, which is ulcerated at the edges, (the effects of the calomel.) Bowels still relaxed; much thirst, and dry tongue. The symptoms assuming rather a typhoid character.

℞. Acid. Sulph. dil. ʒj.; Træ. Cinnamon. comp. ʒiij.; Sp. Æther. Sulph. ʒvj.; Mist. Camph. ℥vij. M. capiat coch. larg. iij. quartâ quâque horâ.—Cont[r] Lotio.

Vesperi.—℞. Ext. Hyosciam., Pulv. Ipecac. comp. āā gr. v. M. fiant pil. ij, horâ somni sumendæ.

29th.—Has passed a quieter night, but complains of the lightness of her head, and the soreness of her tongue.

Cont[r] medicamenta.

Until yesterday, her diet has been confined to tea and gruel; she has since taken some broth.

Vesperi.—Has been out of bed frequently during the day; quite incoherent in her speech; very restless; is not able to identify persons.

℞. Acetat. Morphiæ gr. ss. stat. sumend.

The above having no sedative effect, after a lapse of two hours she took the following—

℞. Ext. Hyosciam. ℈ss. in pil. iij. dividend. statim sumendas.

30th.—Has had some comfortable sleep since.

Two A.M.—Still complains of her head; the ulceration of her tongue very troublesome.

Applic$^{r}$ Emp. Canth. capiti raso.—℞. Mist. Camph. ℥jss. fiat haustus quartâ quâque horâ sumendus.—Repet$^{r}$ pilulæ Hyosciam. vesperi.

31st.—Has slept comfortably, and is much better upon the whole, except her tongue, the soreness of which prevents her taking solid food.

℞. Dec. Cinchonæ ℥jss. fiat haustus quartis horis sumendus.

From this time she continued to improve daily, and soon became convalescent.

*Alcester; March 30th,* 1287.

